# Effect of aging on biomarkers and clinical profile in Parkinson’s disease

**DOI:** 10.1007/s00415-025-13384-7

**Published:** 2025-09-24

**Authors:** Giulia Di Lazzaro, Federico Paolini Paoletti, Giovanni Bellomo, Tommaso Schirinzi, Piergiorgio Grillo, Guido Maria Giuffrè, Martina Petracca, Anna Picca, Nicola Biagio Mercuri, Lucilla Parnetti, Paolo Calabresi, Anna Rita Bentivoglio

**Affiliations:** 1 Neurology Unit, Fondazione Policlinico Universitario Agostino Gemelli IRCCS, Largo Agostino Gemelli 8, 00168 Rome, Italy; 2 Università Cattolica del Sacro Cuore, Rome, Italy; 3 Section of Neurology, Department of Medicine and Surgery, University Hospital of Perugia, Perugia, Italy; 4 Neurology Unit, Department of Systems Medicine, University of Rome “Tor Vergata”, Rome, Italy; 5 Department of Brain and Behavioral Sciences, University of Pavia, Pavia, Italy; 6 IRCCS Mondino Foundation, Pavia, Italy; 7 Department of Medicine and Surgery, LUM University, 70010 Casamassima, Italy

**Keywords:** Ageing, Fluid biomarkers, Parkinson’s disease

## Abstract

**Background:**

Parkinson’s disease (PD) has different progression rates and disease characteristics according to age of onset, the younger being less cognitively affected and experiencing more motor fluctuations. We explored different pathophysiologic mechanisms underlying PD in patients of different ages independently from disease duration, through CSF biomarkers.

**Methods:**

Patients with clinically established diagnosis of PD underwent clinical evaluation through validated clinical scales (MDS-UPDRS, NMSS, MoCA, WOQ, QUIP, UDysRS). CSF inflammatory (YKL-40, TREM-2) and neurodegeneration (A-Beta42 and 40, t-Tau, p-Tau, NfL, Neurogranin, alpha-synuclein) biomarkers were analyzed.

**Results:**

95 PD patients were recruited, among whom 43 were younger than 66 years old, and 52 older. Age strongly correlated with neurofilament CSF levels, both light and heavy chain, with YKL-40 and with tau species. Younger and older patients showed different biomarker profiles. Younger patients showed significantly lower levels of inflammatory molecules (YKL-40), of degeneration biomarkers (tau species, neurofilament light and heavy chains), independently from disease duration. Clinically, younger patients had better scores at MDS-UPDRS parts I and III and were more prone to develop motor fluctuations and impulse control disorders.

**Conclusions:**

Our data support the hypothesis that PD has different underlying biological features in younger and older subjects. Older subjects may have a broader spectrum of disease mechanisms, reflected in the higher prevalence of amyloid pathology and neurodegeneration, which could underlie the worse cognitive performances and lower dyskinesia burden. They could therefore necessitate a wider array of treatment strategies along with dopaminergic supplementation. Consequently, some of these biomarkers hold promise in refining treatment approaches in PD.

## Introduction

Clinical evidence indicates that patients with Parkinson’s disease (PD) may have very different progression rates and disease characteristics [1]. PD is a common neurodegenerative disease of older adults [2], yet its diagnosis is increasingly made in younger subjects. Younger individuals diagnosed with idiopathic PD may show a milder disease course, characterized by longer disease duration prior to the development of signs and symptoms typical of the advanced stage of the disease, such as gait and balance issues or cognitive disturbances. Conversely, younger age is a risk factor for developing motor fluctuations, levodopa-induced dyskinesia (LID), and impulse control disorders (ICD) [3]. However, the biological substrate for these clinical differences has been rarely investigated [4]. A post-mortem study demonstrated a lower Lewy bodies score in younger patients although significant clinical disparities manifest primarily in the early to middle phases of the disease, where pathological data remain scarce [5]. Exploring the protein profile of cerebrospinal fluid (CSF) could provide insights into the pathophysiology of PD and its association with brain aging at different stages of the disease [4, 6]. Several studies have investigated the diagnostic role of CSF biomarkers, such as α-synuclein and neurofilaments light chain (NfL), in differentiating idiopathic PD and atypical parkinsonism, and their prognostic role [6, 7]. Moreover, recent evidence suggests a higher inflammatory activity in early disease stage, with subsequent attenuation [8]. Furthermore, neurodegeneration-associated markers, such as Alzheimer’s disease (AD)-related molecules and neurogranin hold prognostic significance, particularly in cognitive outcomes for people with MCI and PD [9]. Accumulating evidence supports the integration of multiple markers into a comprehensive diagnostic and prognostic model, surpassing the efficacy of single-molecule profiling [6]. However, the effect of age on CSF biomarkers, regardless of disease duration, is still lacking. This is particularly relevant to tailor treatment and assess their disease-modifying effect. Therefore, the present study aimed to evaluate a panel of different mediators in the CSF to verify their potential as biomarkers and elucidate the varying pathophysiology underlying PD across different age groups. Moreover, correlations between clinical and neurochemical data were also evaluated. We decided to focus on the early–middle phase of the disease because we believe this is the most promising phase for disease-modifying treatments and also the stage where clinical differences emerge.

## Materials and methods

### Patients’ enrollment and evaluation

Patients with a clinically established diagnosis of PD were consecutively enrolled during routine clinical practice at three Italian centers (Policlinico Universitario Agostino Gemelli, Policlinico Tor Vergata and Ospedale Santa Maria della Misericordia, Perugia) between July 2020 and December 2021.

Inclusion criteria were: (1) Clinically established diagnosis of PD according to the Movement Disorders Society (MDS) diagnostic criteria [10]; (2) Evidence of dopaminergic denervation at neuroimaging or clinical follow-up of at least 3 years; (3) Absence of red flags according to the MDS diagnostic criteria for PD; (4) Age between 30- and 80-year-old; (5) Symptom onset between 3 and 10 years before enrollment; (6) Absence of known pathogenic variants in genes associated to PD. Patients with Montreal cognitive assessment (MoCA) < 24, Hoehn and Yahr staging > 3, or pregnant were excluded from the study. Furthermore, the presence of any systemic inflammatory or oncological disease was an exclusion criterion. Finally, all patients carrying known pathogenic variants in genes associated with PD were excluded. Specifically, *GBA1* and *LRRK2* variants are routinely screened, and ,in case of a negative result, a panel and MLPA for *PRKN*, *PINK1*, *DJ-1* and *SNCA* was performed. This study was approved by the local ethical committee of each center and informed written consent was signed by all patients enrolled. This study was carried out according to the Declaration of Helsinki.

The clinical and pharmacological history was collected, levodopa-equivalent daily dose (LEDD) was calculated [11] and patients were examined by means of the following clinical scales by a neurologist expert in movement disorders, in the best ON-medication state:

- Unified Parkinson’s Disease Rating Scale—Movement Disorder Society (MDS-UPDRS), parts I–II–III–IV, to evaluate various aspects of PD, including non-motor and motor experiences of daily living and motor complications;
- Hoehn and Yahr scale (H&Y), to assess disease stage;
- Non-motor Symptoms Scale (NMSS) which measures the severity and frequency of non-motor symptoms across nine dimensions: cardiovascular, sleep/fatigue, mood/cognition, perceptual problems, attention/memory, gastrointestinal, urinary, sexual function, and miscellany;
- Unified dyskinesia rating scale (UDysRS), a scale to assess the LID presence and severity;
- Wearing off questionnaire (WOQ), a scale to assess presence and severity of motor and non-motor wearing off signs and symptoms;
- Questionnaire for Impulsive-Compulsive Disorders in Parkinson’s Disease (QUIP), a scale to assess ICD
- MoCA, as screening measure for cognitive functioning. This scale was administered by a MoCA certified evaluator.

### Sample collection and storage

Lumbar puncture was be performed according to international guidelines and a standardized procedure [12]. Briefly, 10–12 mL of CSF was collected in sterile polypropylene tubes (Sarstedt, code: 62.610.201) and centrifuged at room temperature for 10 min (2000×*g*) no longer than 15 min after collection. Aliquots (0.5 mL) were frozen at − 80 °C and stored until analysis. CSF samples with blood contamination were excluded.

### Sample analysis

Biomarkers measured reflected different pathophysiological pathways: inflammation, AD-related pathology, neurodegeneration, and synaptopathy. The levels of YKL4 [13], a marker of astrocyte activation, and sTREM-2 [14], a cell surface receptor important for modulation of microglia immune response, were assayed using commercially available ELISA kits (Human TREM2, Abcam, Cambridge, United Kingdom, and YKL-40 with Human Chitinase 3-like 1 Quantikine, R&D Systems, Minneapolis, MN). With respect to AD-related biomarkers [15], amyloid-β42 and amyloid-β40 (Aβ42 and Aβ40), phosphorylated tau (p-tau), and total tau (t-tau) were measured using Lumipulse G-600-II fully automated chemiluminescent enzyme immunoassay system (Fujirebio Europe, Gent, Belgium), while alpha-synuclein by means of high sensitive ELISA kits (Bio-Techne, San Jose, CA, USA). The levels of neurofilaments, both light (NfL) and phosphorylated heavy (pNfH) chain, a marker of neurodegeneration [16], were determined by commercially available kits on an ELLA™ automated immunoassay system (Bio-Techne, San Jose, CA, USA) according to the manufacturer’s instructions. As biomarker reflecting synaptopathy, we considered neurogranin [17] which is a calmodulin-binding postsynaptic protein, involved in synaptic signaling, plasticity, long-term potentiation and memory consolidation; it was measured using a commercially available ELISA kit (Neurogranin, Trunc P75, Euroimmun, Lubeck, Germany).

### Statistical analysis

Statistical analysis was performed using SPSS v. 28 for Mac. Continuous variables were reported as means, standard deviations, and ranges. Categorical variables were shown as counts and percentages. Continuous variables were tested for normality with Shapiro–Wilk test. Differences among clinical variables were tested with chi-square test for dichotomic variables and with Student’s *t* test for continuous ones. Differences in biomarkers levels in different patients’ groups according to age (younger patients < 66 years old and older patients > 65 years old [18]) were tested by ANCOVA with disease duration and sex as covariates. Bonferroni correction for multiple testing was applied when applicable. Spearman’s *r* correlation coefficients of biomarkers concentrations were calculated. *p* < 0.05 was chosen as the minimum level of statistical significance.

## Results

We enrolled 95 patients with a clinically established diagnosis of PD. Their clinical–demographical data are shown in Table 1. In summary, patients, with a male prevalence (*M*/*F* = 64/31), had a mild to moderate parkinsonism and a disease duration of 7 ± 2 years. 52 patients were elderly patients (> 65 years old), while 43 were adults (< 65 years old). All patients were on Levodopa, alone or in combination of other dopaminergic therapy, for a mean of 683 ± 367 mg of LEDD. Almost half (44/95) experienced motor fluctuations of whom 32 reported LID. There were significant clinical differences in younger and older patients. In particular, younger patients had better scores at MDS-UPDRS parts I and III (Table 1) and a bigger proportion of younger patients experienced motor fluctuations and ICD (respectively 26/43 vs. 18/52, *p* = 0.021 and 20/43 vs. 12/52, *p* = 0.01).

**Table 1:** Clinical–demographical characteristics of PD patients

	PD patients (*n* = 95)	Younger patients (*n* = 43)	Older patients (*n* = 52)	*p* value
Age	**65 ± 9** **years**	**56.1 ± 6.2** **years**	**70.5 ± 4.2** **years**	**< 0.001**
SEX (*M*/*F*)	**64/31**	**33/10**	**31/21**	**0.084**
Age of onset	**55 ± 9** **years**	**47.3 ± 6.3** **years**	**62.3 ± 6.7** **years**	**< 0.001**
BMI	26.5 ± 3.3	26.1 ± 3.3	26.9 ± 3.3	0.191
Disease duration	7 ± 2 years	7.5 ± 3.4 years	6.8 ± 2.4 years	0.144
LEDD	683 ± 387 mg	725 ± 304 mg	659 ± 407 mg	0.22
MoCA	26 ± 2	26 ± 1.7	25.5 ± 1.7	0.181
MDS-UPDRS I	**10 ± 6**	**8 ± 5**	**11 ± 6**	**0.011**
MDS-UPDRS II	10 ± 7	9 ± 7	10 ± 6	0.09
MDS-UPDRS III	**29 ± 13**	**26.5 ± 12**	**33 ± 11**	**0.005**
MDS-UPDRS IV	3 ± 4	4 ± 5	2.5 ± 4	0.23
H&Y	2.3 ± 0.6	2 ± 0.5	2.3 ± 0.5	0.47
WOQ	10 ± 8	11.5 ± 8	10 ± 7	0.166
UDysRS	7 ± 14	8.8 ± 16	6.5 ± 13	0.4
Motor fluctuations (yes/no)	**44/51**	**26/17**	**18/34**	**0.021**
QUIP	2 ± 1	1.1 ± 1.4	0.5 ± 1	0.068
ICD (yes/no)	**32/63**	**20/23**	**12/40**	**0.01**

*M* male, *F* female, *BMI* body mass index, *LEDD* levodopa equivalent daily dose, *MoCA* Montreal Cognitive Assessment, *MDS-UPDRS* Movement Disorders Society-Unified Parkinson’s Disease Rating Scale, *H&Y* Hoehn and Yahr staging, *WOQ* wearing off questionnaire, *UDysRS* unified dyskinesia rating scale, *QUIP* questionnaire for impulsive-compulsive disorders in Parkinson’s disease, *ICD* impulse control disorder. In bold, significant results

**Fig. 1:**
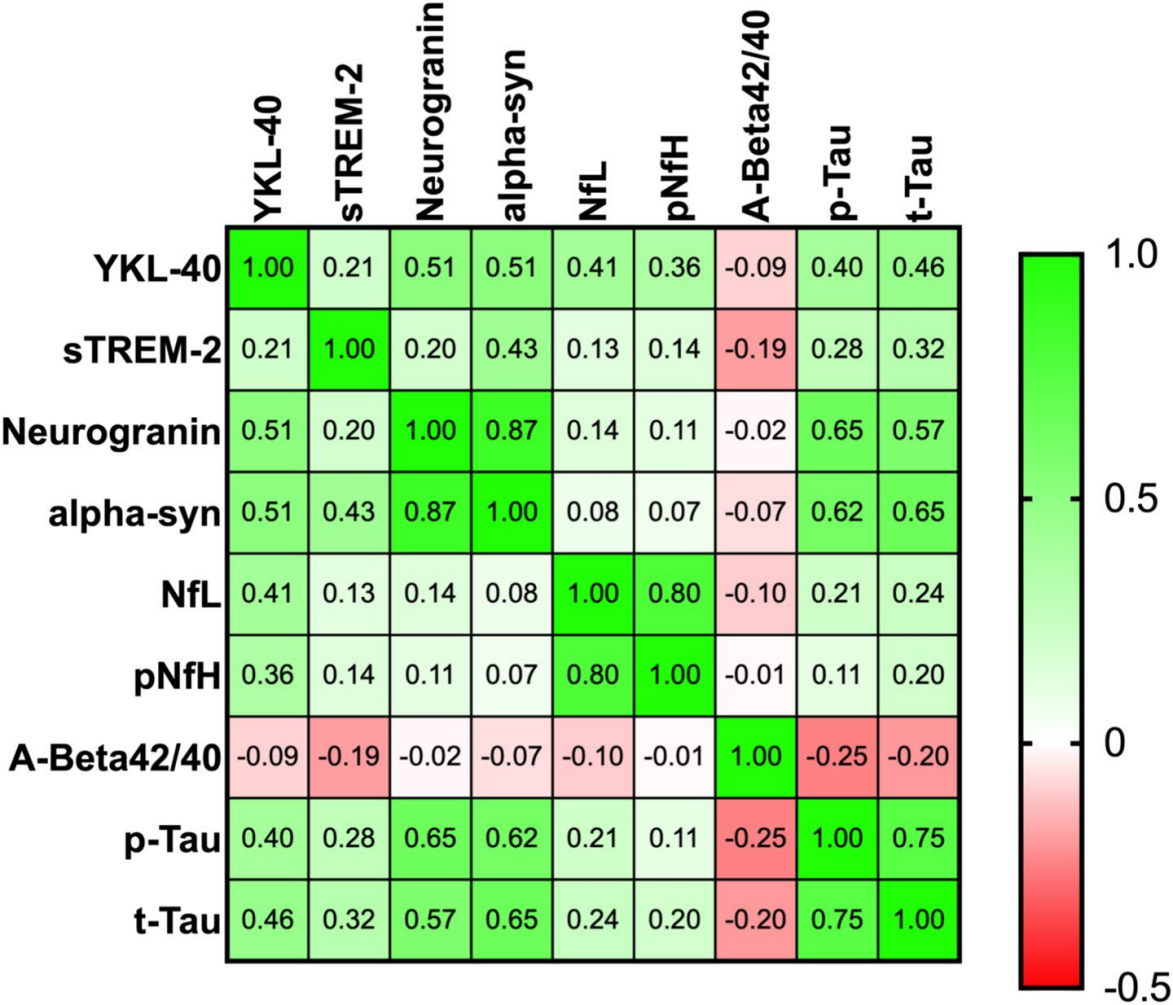
Heath map showing correlations among CSF biomarkers

With respect to CSF biomarkers, Table 2 shows mean ± standard deviation of their levels in our study population. CSF biomarkers levels were similar among the three cohorts from the three recruiting centers (data not shown). A correlation analysis between biomarkers showed several strong and significant correlations as expected (Fig. 1). In particular, biomarkers reflecting the same pathophysiological process had the highest correlations. Aβ42 and Aβ40 correlated well with each other, and t-Tau and p-Tau also correlated very strongly, and both correlated with neurogranin levels. With respect to clinical outcomes, as expected, neurofilaments, both light and heavy chains, positively correlated with MDS-UPDRS III score (respectively *p* = 0.033, *r* = 0.223 and *p* = 0.029, *r* = 0.23). NfL also positively correlated with MDS-UPDRS I scores (*p* = 0.12, *r* = 0.297) as well as Abeta42/40 (*p* = 0.14, *r* = 0.299). QUIP scores correlated with Abeta42/40 (*p* < 0.001, *r* = 0.403) and inversely correlated with t-Tau levels (*p* = 0.13, *r* = 0.307).

**Table 2:** CSF biomarkers level in the study population, correlations between CSF biomarkers levels and age (*p < 0.05; **p < 0.001), and differences between younger and older patients, corrected for disease duration and sex

	PD patients	Correlation with age	Younger patients (*n* = 43)	Older patients (*n* = 52)	*p* value
YKL-40 (ng/mL)	132.37 ± 46.8	.400**	116.74 ± 38.82	152.34 ± 72.35	**0.043**
Neurogranin (pg/mL)	269.8 ± 149.14	0.183	234.52 ± 103.44	310.42 ± 193	**0.038**
NfL (pg/mL)	885.68 ± 457.5	.626**	802.83 ± 662.62	1191.79 ± 1011.45	**< 0.001**
pNfH (ng/mL)	320 ± 137	.533**	269.54 ± 131.39	379.05 ± 183.31	**< 0.001**
t-Tau (ng/mL)	240.8 ± 118.7	.293**	199.31 ± 84.16	296.14 ± 165.09	**0.02**
p-Tau (ng/mL)	33,88 ± 13.47	.317**	29.9 ± 11.73	40.55 ± 22.06	**0.011**
sTREM2 (ng/mL)	39.72 ± 15.73	0.174	38.72 ± 18.01	40.05 ± 13.79	0.85
Alpha-syn (pg/mL)	1743.25 ± 611.4	0.09	1708.62 ± 591.8	1773.64 ± 632.6	0.285
Aβ42/40	0.12 ± 0.04	0.009	0.115 ± 0.043	0.120 ± 0.044	0.5

**Fig. 2:**
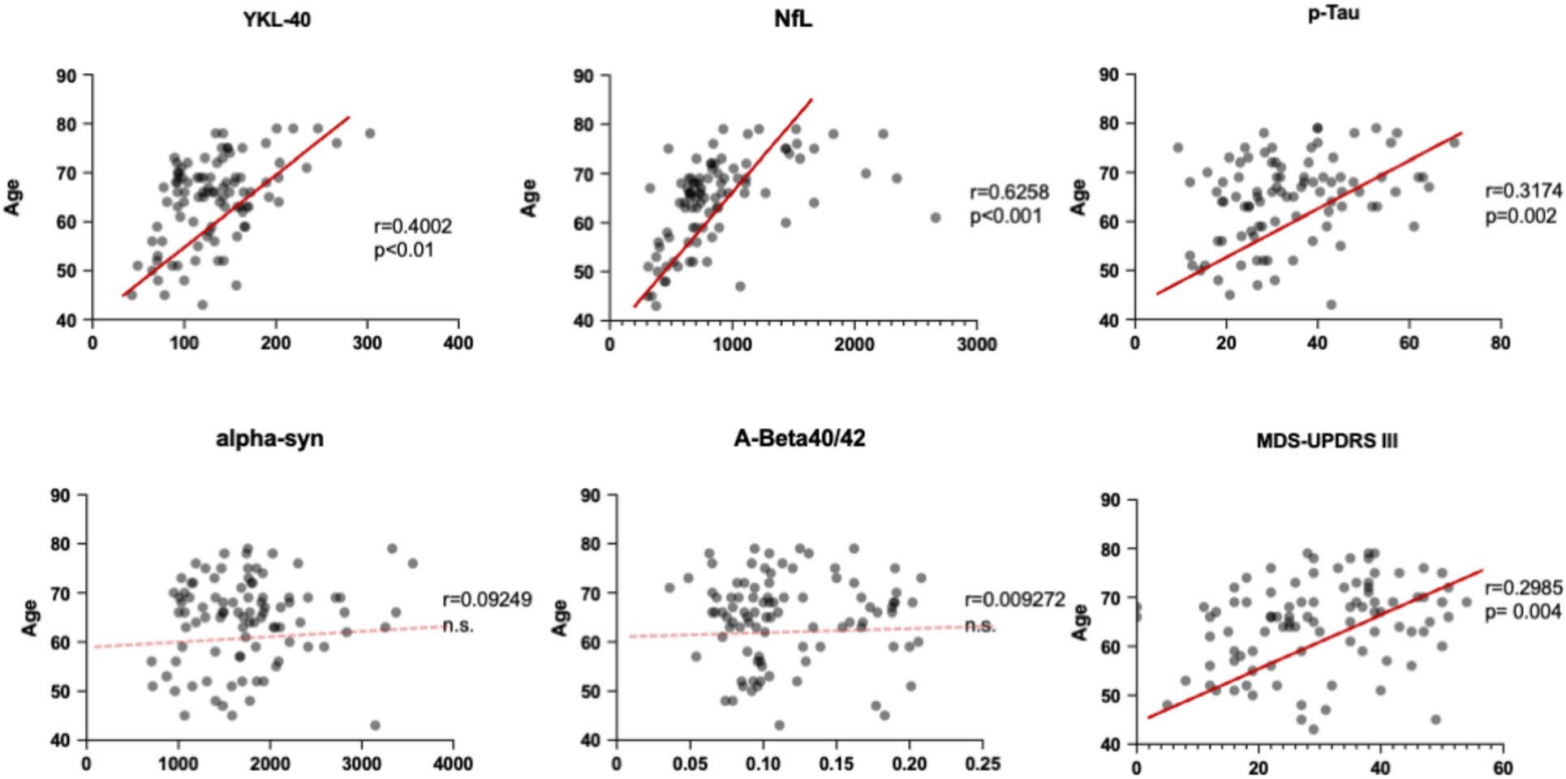
Correlations between age and CSF biomarkers and MDS-UPDRS III

**Fig. 3:**
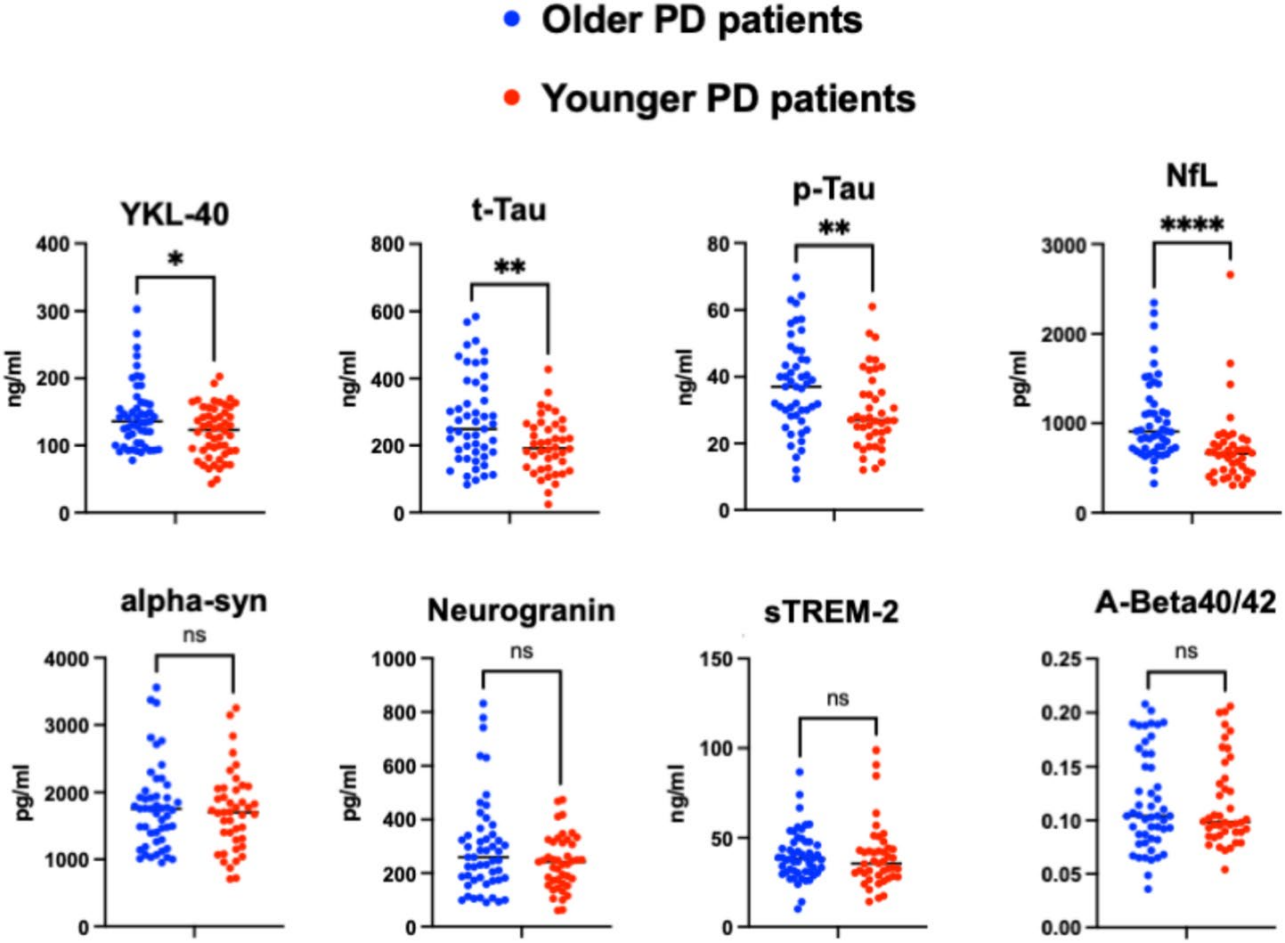
CSF biomarkers differences in younger and older PD patients

Regarding the relationship between biological profile and aging, as shown in Table 2, age strongly correlated with neurofilament CSF levels, both light and heavy chains (respectively *r* = 0.626 and *r* = 0.533, *p* < 0.001), with YKL-40 (*r* = 0.4, *p* < 0.001) and AD-related pathology biomarkers, in particular with tau species (p-Tau *r* = 0.317 and t-Tau *r* = 0.293, *p* < 0.001) (Table 2, Fig. 2). This was reflected in significant differences between younger and older patients (Table 2, Fig. 3) in chosen biomarkers levels. In particular, younger patients showed significantly lower levels of inflammatory molecules (YKL-40), of biomarkers reflecting axonal damage (NfL, pNfH) and AD-related molecules (t-Tau and p-Tau), independently from disease duration and sex.

## Discussion

To the best of our knowledge, this is the first study exploring the relationship between aging and CSF biomarkers in people with PD. Here, we demonstrate that younger PD patients have lower levels of markers of neurodegeneration, AD-related, and inflammatory in the CSF compared to older PD patients. It is established that age is an independent and major risk factor for cognitive decline and parkinsonism, with particular susceptibility above 70 years of age [2, 19]. Moreover, it is well known that younger-onset PD is associated with a longer disease course, a higher rate of motor fluctuations, dyskinesia and, in comparison with older patients, preservation of cognition [3, 20]. In retrospective studies made in advanced stages of PD, it was shown that younger patients had taken a longer time to show motor and cognitive decline [5]. Neuropathological analysis revealed that younger patients show less Lewy bodies pathology, as well as lower amyloid burden and neurofibrillary tangles as compared to older ones. However, the pathophysiological features underlying the early–middle stages of the disease remain unclear. Our data support the hypothesis that younger PD patients could have a more preserved “brain circuitries integrity”, mirrored by a higher concentrations of non-specific markers of neurodegeneration in the older ones [21].

Functional and structural differences associated with brain aging in people with PD could independently contribute to clinical differences between younger and older individuals, irrespective of disease duration. Age-related alterations in basal ganglia circuits, encompassing neurochemical, synaptic functions, and structural pathologies, may underlie varying clinical presentations of nigro-striatal dopaminergic deficits across different age groups. This could explain the more preserved “brain circuitries integrity” in younger patients, potentially explaining reduced motor impairment and superior cognitive function compared to their older counterparts. The same reasons could explain why younger people are more prone to developing other disease-related complications, such as motor fluctuations and LIDs. LIDs are thought to be the expression of an aberrant plasticity which tries to compensate the lack of dopaminergic input from nigrostriatal neurons [22, 23]. Indeed, a reasonable pathophysiological hypothesis is that this aberrant plasticity requires a minimal threshold of neuronal and synaptic integrity to be significant and have a clinical correlate.

Another factor which could play a role is the genetic background, which may more commonly underlie younger-onset PD and be responsible of a peculiar pathogenic mechanisms underlying the disease [24]. However, in our cohort, patients were tested for the most common pathogenic variants known to be associated with PD and they were excluded. This does not account for the cumulative effect of common variants or contribution of novel contributing genes and will have to be addressed by future studies.

A possible hypothesis resulting from this study is that a combination of multiple mediators reflecting alterations in neuronal circuits and neurodegeneration, such as NfL, neurogranin, and tau proteins, indicates loss of brain integrity which in turn reflects a worse clinical progression in PD patients. These mediators could be therefore useful biomarkers of the beneficial effects of non-dopaminergic drugs as well as of non-pharmacological approaches, such as physical exercise and non-invasive brain stimulation, in the early–middle disease stages. They could also help in the early identification of patients with a higher risk of a faster progression, who could benefit from early rehabilitation interventions.

This study has several limitations. First, the lack of a control cohort, which could have supported further the idea that aging is the prominent determinant of the biochemical CSF profile. Nonetheless, associations for certain biomarkers are established from historical cohorts [16, 25–27]. Furthermore, the aim of our study was not to identify diagnostic markers but to gain pathophysiological insights in a homogeneous cohort of PD patients.

Moreover, CSF biomarkers dosage lacks spatial specificity when compared to pathological studies. Therefore, we cannot infer on which area is more susceptible to aging in PD patients. Further studies with a coupling with structural and functional neuroimaging may help overcome this limitation. Another possible limitation is the selection bias since the enrolling centers are tertiary centers. However, we tried to choose inclusion criteria which could minimize the presence of complex confounding cases, including only patients in a limited age range and with an early uncomplicated PD. Finally, some of the correlation findings may be due to chance and therefore replication of such results in larger population is needed.

In conclusion, our data support the hypothesis that PD has different underlying biological features in younger and older subjects. Older subjects may have a broader spectrum of disease mechanisms, necessitating a wider array of treatment strategies and distinct neuromodulation protocols. Consequently, some of these biomarkers hold promise in refining treatment approaches in PD beyond dopamine supplementation. Further data, also from pathological studies, are needed to support this perspective.

## Data Availability

The data that support the findings of this study are available from the corresponding author upon reasonable request.
